# Mortality among patients with sepsis associated with a bispectral electroencephalography (BSEEG) score

**DOI:** 10.1038/s41598-021-93588-9

**Published:** 2021-07-09

**Authors:** Takehiko Yamanashi, Pedro S. Marra, Kaitlyn J. Crutchley, Nadia E. Wahba, Johnny R. Malicoat, Eleanor J. Sullivan, Cade C. Akers, Catherine A. Nicholson, Felipe M. Herrmann, Matthew D. Karam, Nicolas O. Noiseux, Koichi Kaneko, Eri Shinozaki, Masaaki Iwata, Hyunkeun Ryan Cho, Sangil Lee, Gen Shinozaki

**Affiliations:** 1 Department of Psychiatry and Behavioral Sciences, Stanford University School of Medicine, 3165 Porter Drive Room 2175, Palo Alto, CA 94304 USA; 2 Department of Psychiatry, University of Iowa Carver College of Medicine, Iowa City, IA USA; 3 Department of Neuropsychiatry, Faculty of Medicine, Tottori University, Yonago, Tottori Japan; 4 Department of Orthopedic Surgery, University of Iowa Carver College of Medicine, Iowa City, IA USA; 5 Department of Internal Medicine, University of Iowa Carver College of Medicine, Iowa City, IA USA; 6 Department of Biostatistics, University of Iowa College of Public Health, Iowa City, IA USA; 7 Department of Emergency Medicine, University of Iowa Carver College of Medicine, Iowa City, IA USA

**Keywords:** Electroencephalography - EEG, Diagnostic markers, Predictive markers, Bacterial infection

## Abstract

We have previously developed a bispectral electroencephalography (BSEEG) device, which was shown to be effective in detecting delirium and predicting patient outcomes. In this study we aimed to apply the BSEEG approach for a sepsis. This was a retrospective cohort study conducted at a single center. Sepsis-positive cases were identified based on retrospective chart review. EEG raw data and calculated BSEEG scores were obtained in the previous studies. The relationship between BSEEG scores and sepsis was analyzed, as well as the relationship among sepsis, BSEEG score, and mortality. Data were analyzed from 628 patients. The BSEEG score from the first encounter (1st BSEEG) showed a significant difference between patients with and without sepsis (*p* = 0.0062), although AUC was very small indicating that it is not suitable for detection purpose. Sepsis patients with high BSEEG scores showed the highest mortality, and non-sepsis patients with low BSEEG scores showed the lowest mortality. Mortality of non-sepsis patients with high BSEEG scores was as bad as that of sepsis patients with low BSEEG scores. Even adjusting for age, gender, comorbidity, and sepsis status, BSEEG remained a significant predictor of mortality (*p* = 0.008). These data are demonstrating its usefulness as a potential tool for identification of patients at high risk and management of sepsis.

## Introduction

Sepsis is one of the most common causes of in-hospital death; one in three patients die from this condition^1, 2^. The national figures closely mimic this ratio, with 270,000 deaths out of 1.7 million cases of sepsis each year^2^. Given how frightening these numbers are, early detection of sepsis patients at risk for poor outcome is pivotal for prompt intervention and better outcomes, especially because a septic patient’s condition can deteriorate rapidly. Every hour of delay in prescribing antimicrobial medication to the septic patient results in a 7.6% decrease in survival rate^3^. Thus, early identification of patients with sepsis at risk for poor outcome is a key step to improve the treatment of this devastating condition. However, current criteria (Sepsis-3) requires an interpretation of mental status included in the quick Sequential Organ Failure Assessment (qSOFA)^4, 5^. They defined altered mental status change using Glasgow Coma Scale ≤ 13, which requires a careful exam of the eye, verbal, and motor exam at the bedside. The interrupter reliability of GSC is moderate^6^, so there is a need for more objective measurement.

Many previous studies have investigated the role of electroencephalography (EEG) in the detection of encephalopathy in the context of delirium, and they have reported largely positive associations between EEG change and sepsis-associated encephalopathy (SAE). Based on these findings, those reports recommend the use of EEG to identify brain abnormalities related to sepsis^7–9^, which can precede the other physiological changes noted during sepsis^10^. For instance, a review paper proposes that SAE can be an early identification of sepsis before all the “requirements” for diagnosis are met^11^. Nevertheless, EEG has not been regularly utilized in current clinical practice. This is probably due to the difficulty in implementing EEG for routine use, especially in remote sites such as urgent care clinics or busy emergency department (ED) settings. We speculate that similar to the case of EEG not being commonly used in screening for delirium due to its size, cost, and expertise required for electrode placement and interpretation of results, EEG is also not used for sepsis detection and/or diagnosis^12^.

It is reported that more than 30% of patients with sepsis have SAE^9^, and delirium/encephalopathy and mild EEG slowing seem to be one of its earliest features^10, 11^. As we have previously developed a point-of-care BSEEG method, which was shown to be effective in detecting delirium and predicting patient outcomes^13–15^, in this study, we aimed to apply the BSEEG approach to see if the same EEG-based method can be useful for the identification of patients with sepsis at high risk for poor outcomes.

## Results

### Participant demographics

A total of 628 patients were enrolled in this study. The average patient age was 66.6 years (SD = 15.4), 51.9% were female, and 95.9% were non-Hispanic white per self-report. Five hundred ten (81.2%) patients were sepsis-negative, and 118 (18.8%) patients were sepsis-positive (Table 1). Flow diagram of participants through the study is shown in Supplemental Figure 1.

**Table 1:** Patient characteristics.

Classification	All subjects	Non-sepsis	Sepsis	*p *value
N	628	510	118	
%		81.2	18.8	
Mean age—years	66.6	66.6	66.6	n.s
SD	15.4	15.7	14.3	
Female sex (n)	326	273	53	n.s
%	51.9	53.5	44.9	
**Race**				
White (*n*)	602	488	114	n.s
%	95.9	95.7	96.6	
Other (*n*)	26	22	4	n.s
%	4.1	4.3	3.4	
CCI	3.2	2.9	4.5	***
SD	2.9	2.7	3.0	

Age, sex, and race were not significantly different between sepsis-negative and sepis-positive groups. CCI was significantly different between sepsis negative and sepsis positive groups.

*SD* standard deviation, *CCI*  Charlson Commobidity Index.

***p < 0.001.

### Mortality based on sepsis status

**Figure 1:**
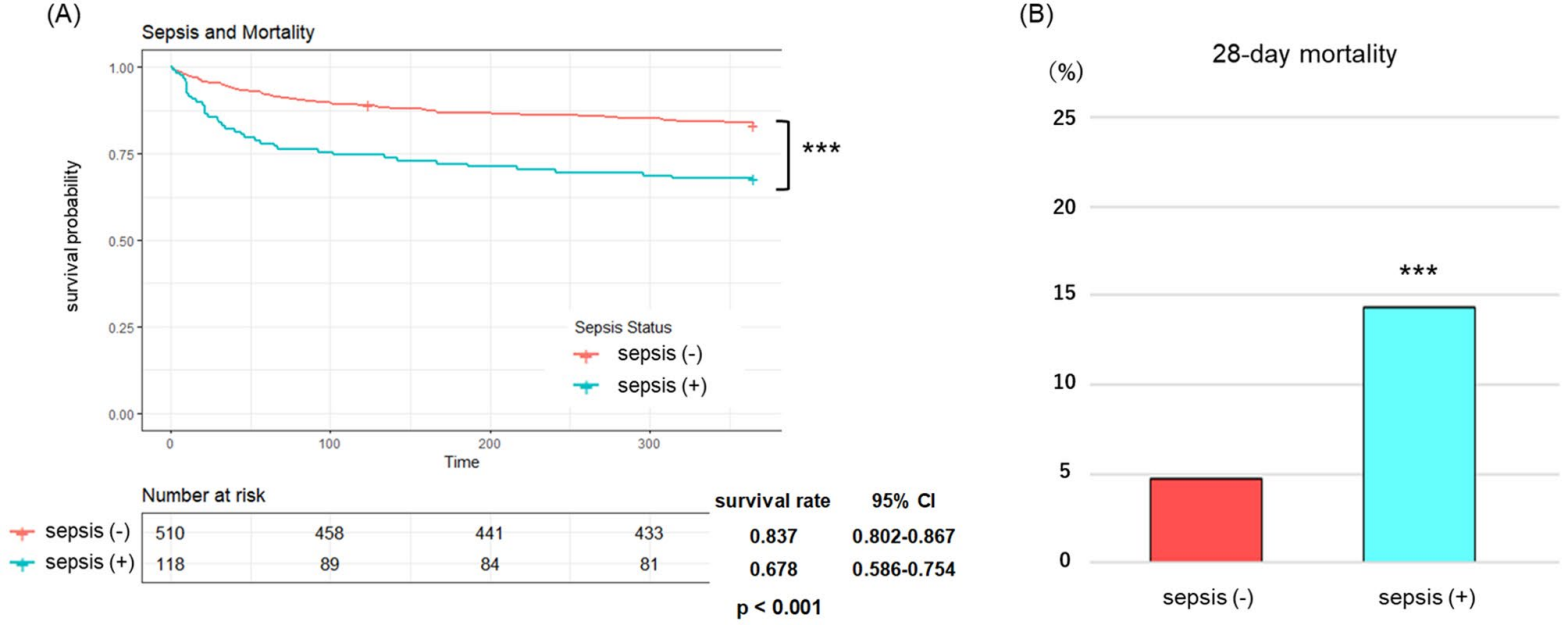
(**A**) Kaplan–Meier survival curve over 365 days by comparing two groups based on whether they had current sepsis or not. Log-rank statistic was performed to assess significance of difference in 365-day mortality. (**B**) 28-day mortality by comparing two groups based on whether they had current sepsis or not. Chi-square test was performed to assess significance of difference in 28-day mortality between the two groups. ***p < 0.001, sepsis (−): sepsis-negative, sepsis (+): sepsis-positive.

First, 365-day mortality was compared between the two groups to confirm that sepsis categorization based on our definition is reasonably accurate by showing that sepsis is associated with high mortality. Patients with sepsis had a lower survival rate than patients without sepsis (0.678 vs 0.837, *p* < 0.001; log-rank analysis) (Fig. 1A). When we focused on 28-day mortality, patients with sepsis had a higher mortality than ones without sepsis as well (14.4% vs 4.7%, *p* < 0.001; Chi-squared test) (Fig. 1B).

### BSEEG score and sepsis status

**Figure 2:**
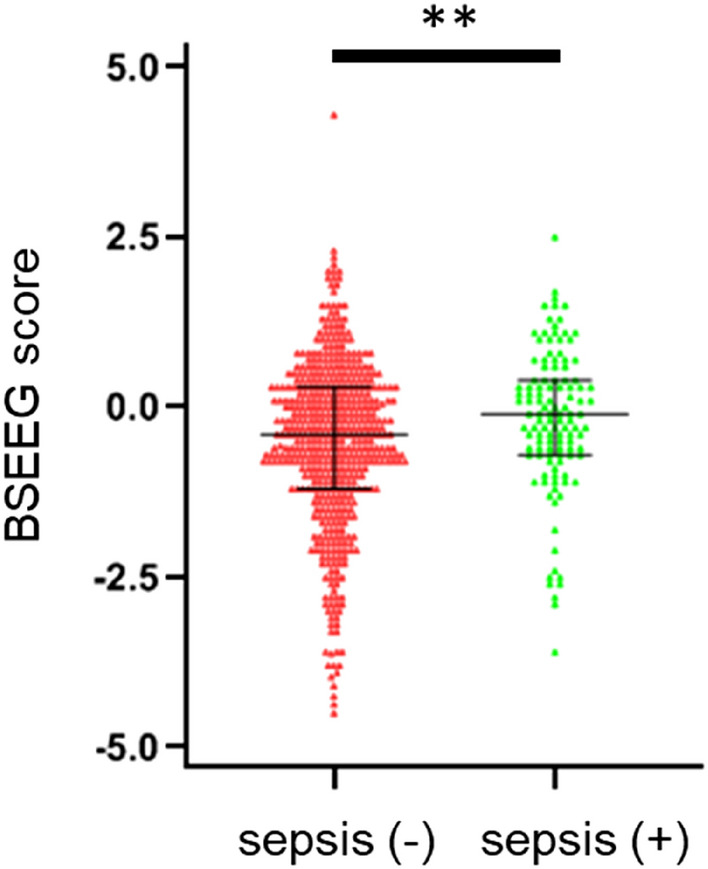
Comparison of 1st BSEEG score among two groups based on sepsis status. The data are presented as scatter plots including median and interquartile range. Mann–Whitney’s *U*-test was performed to compare the two groups. **p < 0.01, sepsis (−): sepsis-negative, sepsis (+): sepsis-positive.

Next, the relationship between sepsis status and the 1st BSEEG score was determined by comparing the statistical differences between the 1st BSEEG score between each group (Fig. 2). Patients with sepsis showed statistically significant higher median 1st BSEEG score (− 0.1) compared to patients without sepsis (− 0.4) (W = 25,053, *p* = 0.0045). The receiver operating characteristic (ROC) curve analysis based on 1st BSEEG and sepsis status is shown in Supplementary Figure 2. The area under the curve (AUC) from the ROC curve was 0.58 (95% CI 0.53–0.64).

### Mortality based on sepsis status and 1st BSEEG score

**Figure 3:**
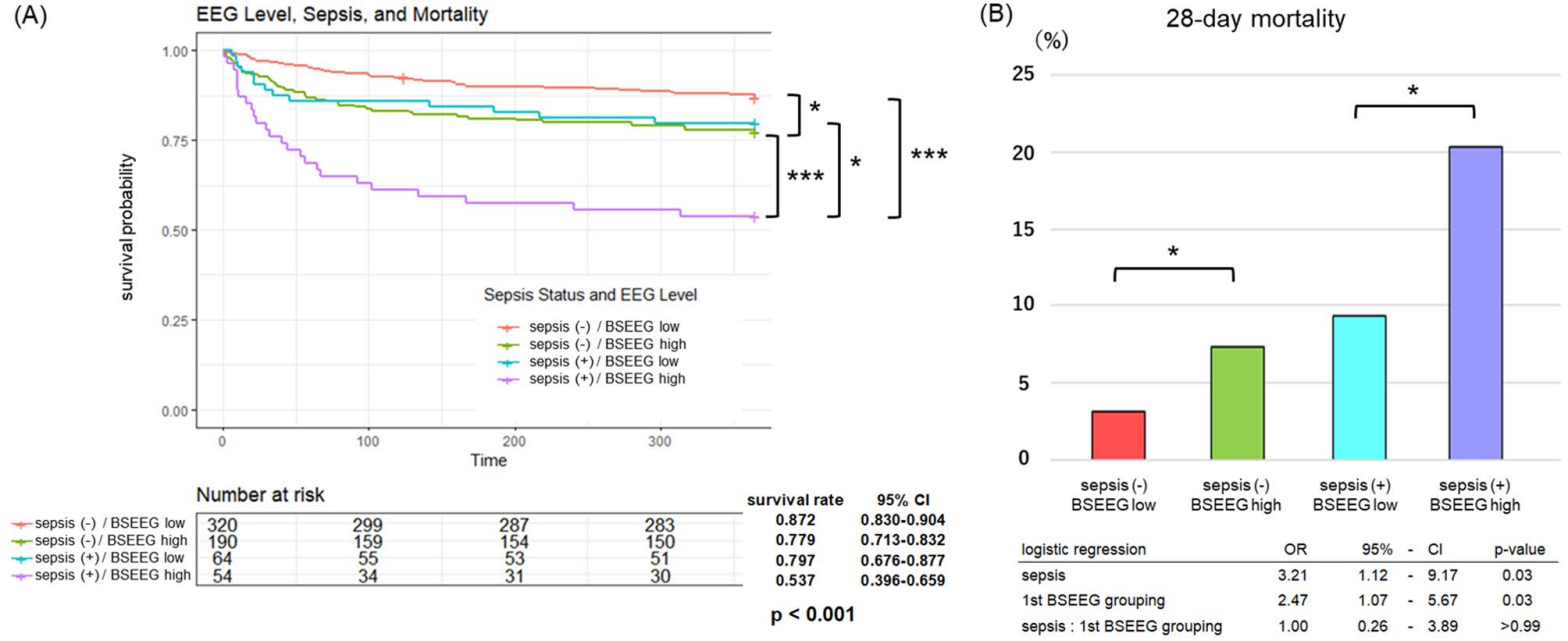
(**A**) Kaplan–Meier survival curve over 365 days by comparing four patient groups based on whether they had current sepsis and/or high BSEEG score (indicative of more slow waves). Log-rank statistic was performed to assess significance of difference in 365-day mortality. (**B**) 28-day mortality by comparing four groups based on whether they had current sepsis or and/or high BSEEG score. Logistic regression including sepsis status, BSEEG grouping, and an interaction between two variables was performed to assess significance of difference in 28-day mortality. *p < 0.05, ***p < 0.001, sepsis (−): sepsis-negative, sepsis (+): sepsis-positive.

When subjects were divided in four groups based on sepsis status (with and without sepsis) and BSEEG score high (more slow waves) or low (fewer slow waves), survival rate was clearly the lowest among those with current sepsis, showing a high BSEEG score (purple) (365-day; 46.3%, 28-day; 20.4%). Compared to them, even among current sepsis patients, if BSEEG is low (blue), mortality was much less (365-day; 20.3%, 28-day; 9.4%). The group without sepsis and showing low BSEEG score (red) showed the least mortality risk (365-day; 12.8%, 28-day; 3.8%). Compared to that, the group without sepsis but showing high BSEEG score (green) showed mortality risk as bad as the group with sepsis but low BSEEG score (365-day; 22.1%, 28-day; 7.4%) (Fig. 3A, B). Cox proportional hazard regression models showed that age, Charlson Comorbidity Index (CCI) score, sepsis status, and BSEEG score significantly related to mortality (Table 2).

**Table 2:** Result of the Cox proportional hazard model.

	HR	95% CI	*p* value
Age	1.03	1.02–1.05	< 0.001***
Sex, female	0.97	0.68–1.39	0.885
CCI	1.17	1.12–1.23	< 0.001***
Current sepsis	1.61	1.08–2.41	0.021*
1st BSEEG score	1.25	1.06–1.48	0.008**

*CCI* Charlson Commobidity Index.

*p < 0.05; **p < 0.01; ***p < 0.001.

### BSEEG score dose-dependent increase of mortality among septic patients

**Figure 4:**
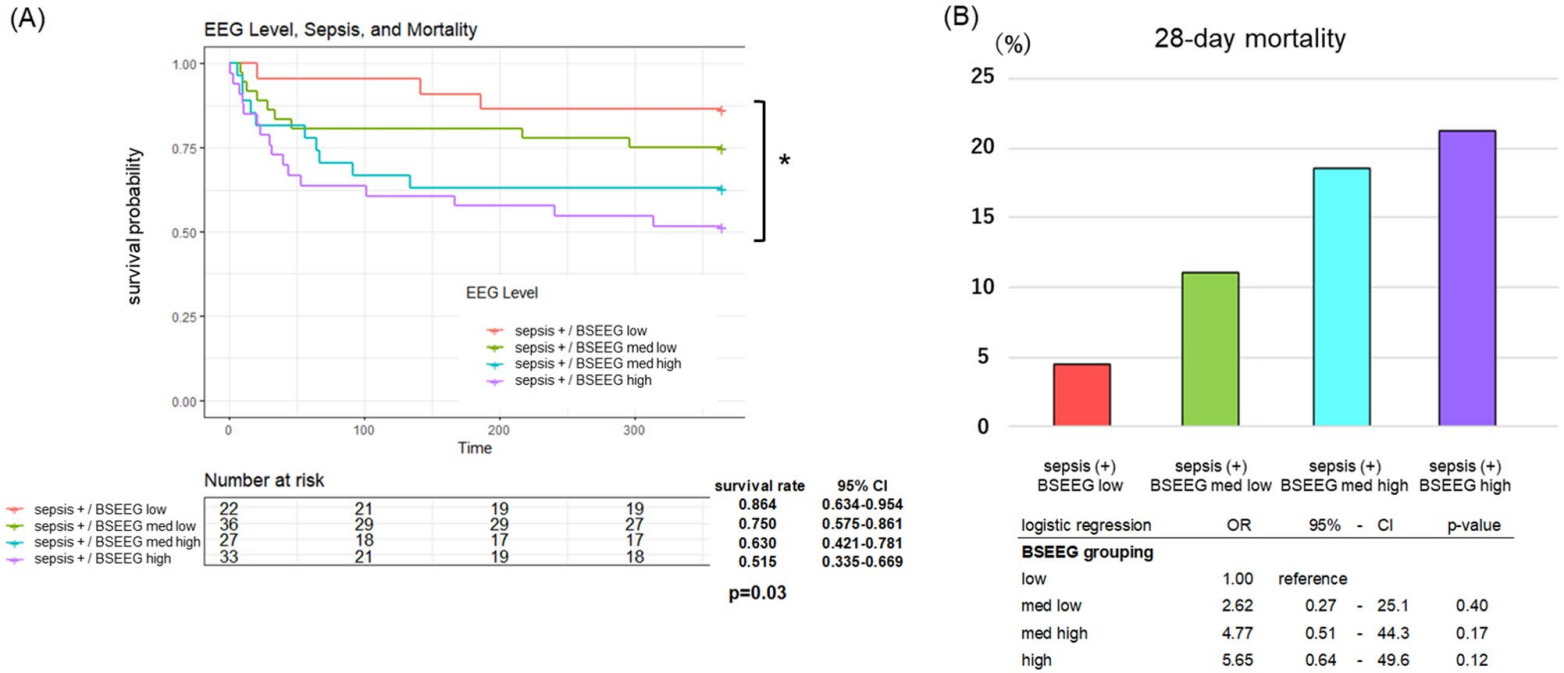
(**A**) Kaplan–Meier survival curve over 365 days by comparing four sepsis-positive patient groups based on BSEEG score. Log-rank statistic was performed to assess significance of difference in 365-day mortality. (**B**) 28-day mortality by comparing four sepsis-positive patient groups based on BSEEG score. Logistic regression including the BSEED score-based grouping to assess significance of difference in 28-day mortality. *p < 0.05, sepsis (+): sepsis-positive.

The data above showed the usefulness of BSEEG score to distinguish high or low mortality risk among sepsis patients. Next, we tested if BSEEG score can differentiate mortality risk more precisely based on BSEEG score, as we have previously shown in the case of delirium subjects^14^. Figure 4 further shows that four grouping based on BSEEG score; high, middle high, middle low, and low, can differentiate mortality risk in a dose-dependent manner of BSEEG score (Fig. 4A, B). Cox proportional hazard regression models showed that age and BSEEG score significantly related to mortality (Table 3).

**Table 3:** Result of the Cox proportional hazard model in patients with sepsis (N = 118).

	HR	95% CI	*p *value
Age	1.04	1.01–1.07	0.009**
Sex, male	0.67	0.35–1.27	0.216
CCI	1.06	0.96–1.16	0.239
1st BSEEG score	1.49	1.06–2.09	0.022*

*CCI* Charlson Commobidity Index.

*p < 0.05; **p < 0.01.

## Discussion

While in previous studies we investigated the association between BSEEG scores and delirium through our novel BSEEG approach^13, 16^, in this present study we applied the BSEEG method to investigate if the BSEEG can be helpful for the care of patients with sepsis. Our data showed that BSEEG scores were higher among the sepsis group compared to the non-sepsis group, although AUC was very small indicating that BSEEG score is not useful for detection of sepsis. However, those with increased BSEEG scores in the sepsis-positive group had a significantly higher mortality rate, suggesting the potential usefulness of BSEEG in the identification of patients with sepsis at high risk of poor outcomes.

Supporting our hypothesis to the limited extent, septic patients had significantly higher 1st BSEEG scores (indicative of more slow waves) (Fig. 2). This is consistent with the literature showing that EEG can potentially detect brain abnormalities in septic patients, thus indicating the presence of SAE^8, 10^. Other studies have also found that lack of EEG reactivity is associated with increased mortality risk^17, 18^ and occurrence of delirium^18^ in patients with sepsis. A review has found conflicting findings, however, of the association between EEG abnormalities and SAE because some studies did not find an incidence of SAE related to background EEG abnormalities^8^. These conflicting results might be due to different criteria used to define encephalopathy. For example, some studies used the CAM-ICU to identify SAE while others did not. Also, diagnosis of SAE’s altered mental status remains subjective no matter how good the instruments/questionnaires are, as administers can change, and interrater reliability cannot be 100%. Thus, we believe that BSEEG scores can be used as a potential tool to capture brain abnormality and/or altered mental status among septic patients by objective measurement. This would be advantageous because one of the criteria for sepsis includes subjectively defined altered mental status. ROC curve, however, was very low and did not show the usefulness of BSEEG method for detection of sepsis (Supplementary Figure 2). It might be partly due to the broad definition of sepsis in this study. It might be possible to demonstrate its detection ability if subjects were limited to septic patients with altered mental status.

On the other hand, our data showed that among septic patients, high BSEEG score (more slow waves) is related to higher mortality risk (Fig. 3). This was shown to be true in a dose-dependent manner even if the groups were divided into four groups (Fig. 4). These high mortality risks in subjects with higher BSEEG scores were observed both in long term period (365 days) and short term period (28 days) (Figs. 3, 4). It is possible that BSEEG can identify septic subjects who are at risk for poor outcome risk through detecting their encephalopathy. Septic patients with encephalopathy have higher mortality than septic patients without encephalopathy, which demonstrates the importance of detecting this devastating condition early^19–21^. Furthermore, because SAE is common in septic patients-up to 70% depending on how SAE is defined^7^—its timely and accurate detection can potentially help diagnose sepsis sooner. At present, however, there is a lack of point-of-care devices that give reliable, objective, and quick results^22^. Our present results suggest that BSEEG method can be a potentially useful tool to identify septic patients who are at risk for poor outcomes including mortality.

Our previous studies showed that the BSEEG approach can detect delirium subjects^13, 15, 16^ and predict poor outcomes^14^. It has also been shown that increased BSEEG score was observed in lipopolysaccharides-induced delirium mouse model^23^. The BSEEG approach presented here is an objective marker of brain dysfunction, as it indicates more slow waves compared to high-frequency waves. Thus, this BSEEG method could potentially be a point-of-care device to assist sepsis patient care practice. The BSEEG device would be more practical in the clinical setting than the standard EEG because it is much smaller, less expensive, does not require a trained technician to place the electrodes, and does not need a specialized electrophysiologist for data interpretation.

Our study has several limitations. First, this study used the same algorithm we developed to calculate the BSEEG scores originally designed to detect delirium, not sepsis. Therefore, it was not a surprise that the current BSEEG score was not showing good performance to detect sepsis. A future goal would be to update the algorithm to be better capable of distinguishing septic patients from non-septic patients. Second, our definition of sepsis may be prone to misclassification as it was not based on specific criteria, and it solely depended on retrospective chart review of electronical medical records (EMRs). Thus there are certain possibilities of false-positive and false-negative cases in our dataset. However, even with such a potential limitation, our grouping showed significant difference in mortality between sepsis and non-sepsis groups, supporting the validity of our grouping of sepsis subjects. Third, this study enrolled patients with sepsis based on a previous cohort used in our delirium studies, which could potentially generate a biased conclusion since sepsis and delirium tend to overlap and are associated. Thus, an independent cohort recruited specifically for sepsis may better serve to evaluate the usefulness of this approach. Other limitations include the study demographics, as 95.9% of patients were non-Hispanic white, and the fact that the study was conducted at a single medical center. However, even with these limitations, our data have shown supporting evidence that the BSEEG approach can be potentially helpful to show differences in septic patients and predict mortality risk.

In summary, we reported that there was an association between BSEEG score and sepsis, although it was not suitable for detection of sepsis. Also, we found that the BSEEG score could predict mortality risk among septic patient group, proving its potential to become a useful clinical tool for the care of septic patients by identifying those at high risk. Our next step would be to test the longitudinal BSEEG score to monitor treatment response for sepsis and encephalopathy.

## Methods

### Design

We conducted a retrospective cohort study with additional chart review using an existing data collected through previous clinical observational studies of delirium at the University of Iowa Hospitals and Clinics (UIHC)^13, 14, 24^. This study was approved by the UIHC Institutional Review Board, and was performed in accordance with relevant guidelines and regulations.

### Study participants

Details of study subjects and enrollment process have been described previously^13, 14, 24^. Inclusion criteria was (1) subjects who were admitted to the emergency department, the orthopedics floor, the general medicine floor, or the intensive care unit in UIHC; (2) subjects whose age were from 18 years or older. Exclusion criteria was (1) subjects whose goals of care were comfort measures only; (2) subjects with droplet/contact precautions; (3) prisoners. Eligible patients were approached to assess their capacity to provide consent and participate. If they were judged not to have the capacity to consent, we obtained consent from a legally authorized representative. We obtained written informed consent from participants or their legally authorized representative after providing a complete description of the study. We analyzed data from subjects enrolled in the study from January 2016 to December 2018.

### Clinical assessment and case definition of sepsis

We collected clinical data as we described previously^13, 14, 24^. Briefly, we obtained demographics information and baseline medical and surgical history from patient interviews and EMRs. We calculated CCI, which indicates severity of comorbidities and is known to be an excellent predictor of mortality^25^. We reviewed each subject using EMRs to capture if there was evidence of sepsis during their follow-up period. Sepsis-positive cases were identified based on clinical documentation of sepsis using a word search strategy for the words “sepsis” or “septic” in subjects’ EMRs during the study period. When such words were found, detailed contexts were reviewed to confirm sepsis cases. For example, if “sepsis” was listed in the EMRs among a list of differential diagnosis, the patient was not considered to be septic. Only when it was mentioned that the patient had been diagnosed with sepsis that we identified the patient as being part of the sepsis-positive category. Sepsis diagnosis was made by the physicians treating those patients while in the hospital. The Sepsis case definition in this study was purely based on the clinician's impression and diagnosis of sepsis, not based on specific criteria. A differential diagnosis including sepsis at the early phase of their admission was not considered sufficient by itself to categorize the patients having sepsis.

### BSEEG data collection and score definition

We collected raw EEG data and calculated BSEEG score as previously described^13–15^. Briefly, EEG was collected using a portable, handheld EEG device (CMS2100, CONTEC, Qinhuangdao, Hebei, China) up to twice daily during the daytime. The patient’s forehead was cleaned with an alcohol swab. Patients were asked to close their eyes and relax their jows. Then, research team members placed electrodes on the center of forehead, on the left and right sides of the forehead (Fp1 and Fp2), and on both sides of the earlobe (A1 and A2) for two-channel recordings. In case of one-channel recording, we used Fp1 and A1 unless that side was not feasible due to a patient’s medical condition or positioning. EEG was recorded for 3–5 min and up to 10 min. The obtained raw EEG data was converted into spectral density plots, and the signal-processing algorithm was used to produce a BSEEG score based on a ratio between low-frequency (3 Hz) power to high-frequency (10 Hz) power^13–15^. Then, the normalized BSEEG score was determined by the number of standard deviations (SDs) from the mean of our accumulated data^14^. Based on this normalization process, average raw BSEEG value is converted as BSEEG score = 0, and raw BSEEG score 1 SD above average becomes BSEEG score = 1. The very first BSEEG score obtained during their study duration was termed “1^st^ BSEEG.” We investigated the relationship between the BSEEG score and subject’s clinical status of sepsis as well as their mortality.

### Assessment of mortality

We assessed all-cause mortality from the study participants based on review of their EMRs and obituary records as previously described^14, 24^. We compared mortality among groups based on status of sepsis and BSEEG score.

### Statistical analysis

All statistical analyses were performed with R^26^. To compare the relationships between the BSEEG score and sepsis, Mann–Whitney’s U-test was performed. The data are presented with scatter plots, median, and interquartile range. ROC curve and AUC were also used to analyze the relationship between BSEEG score and sepsis-positive cases. We used Kaplan–Meier survival curves to visualize time to death. Log-rank statistics were used to assess significance of difference in 365-day mortality. We also compared 28-day mortality. For mortality analysis, we first divided subjects into two groups based on their sepsis status (sepsis-negative or sepsis-positive). Chi-square test was used to assess significance of difference in 28-day mortality between the two groups. We also divided subjects into four groups based on sepsis status, and BSEEG high or low (BSEEG high indicates slower brain waves). We used logistic regression including sepsis status, BSEEG grouping, and an interaction between two variables to assess significance of difference in 28-day mortality. Finally, we divided subjects with sepsis into four groups based on their BSEEG score. We used logistic regression including the BSEED score-based grouping to assess significance of difference in 28-day mortality. *p* values were corrected by Bonferroni method when three or more group were compared. We also used Cox proportional hazard regression models to obtain hazard ratios (HRs) of death up to 365 days from study enrollment controlling for age, sex, CCI, and sepsis status. *p* values < 0.05 were considered significant.

## Supplementary Information

Supplementary Information.
