# Supplementary material for: Mortality among patients with sepsis associated with a bispectral electroencephalography (BSEEG) score
Source: Sci Rep. 2021 Jul 9;11:14211. doi: 10.1038/s41598-021-93588-9 (PMC8270989; doi:10.1038/s41598-021-93588-9)
Supplement: Supplementary file 1 — Supplementary Information. [file 41598_2021_93588_MOESM1_ESM.pdf]

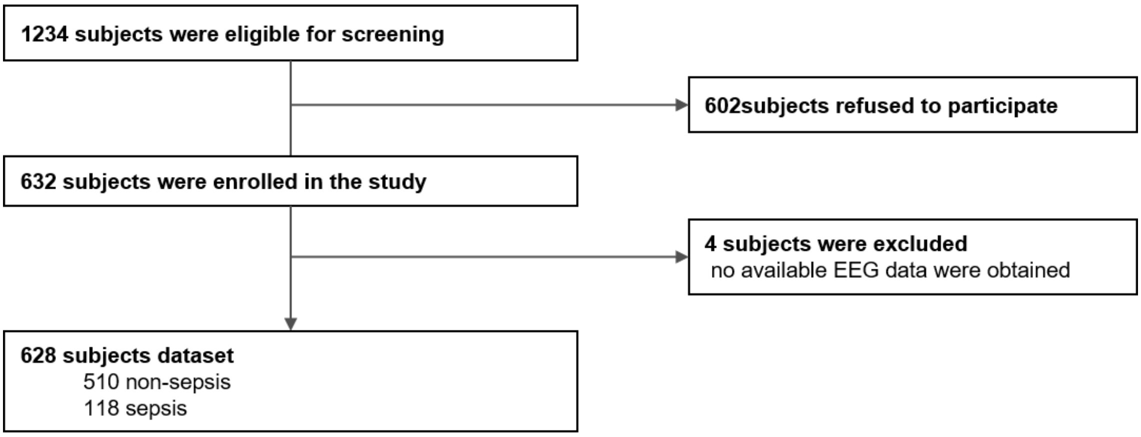

Supplementary Figure 1: Flow of participants through the study.

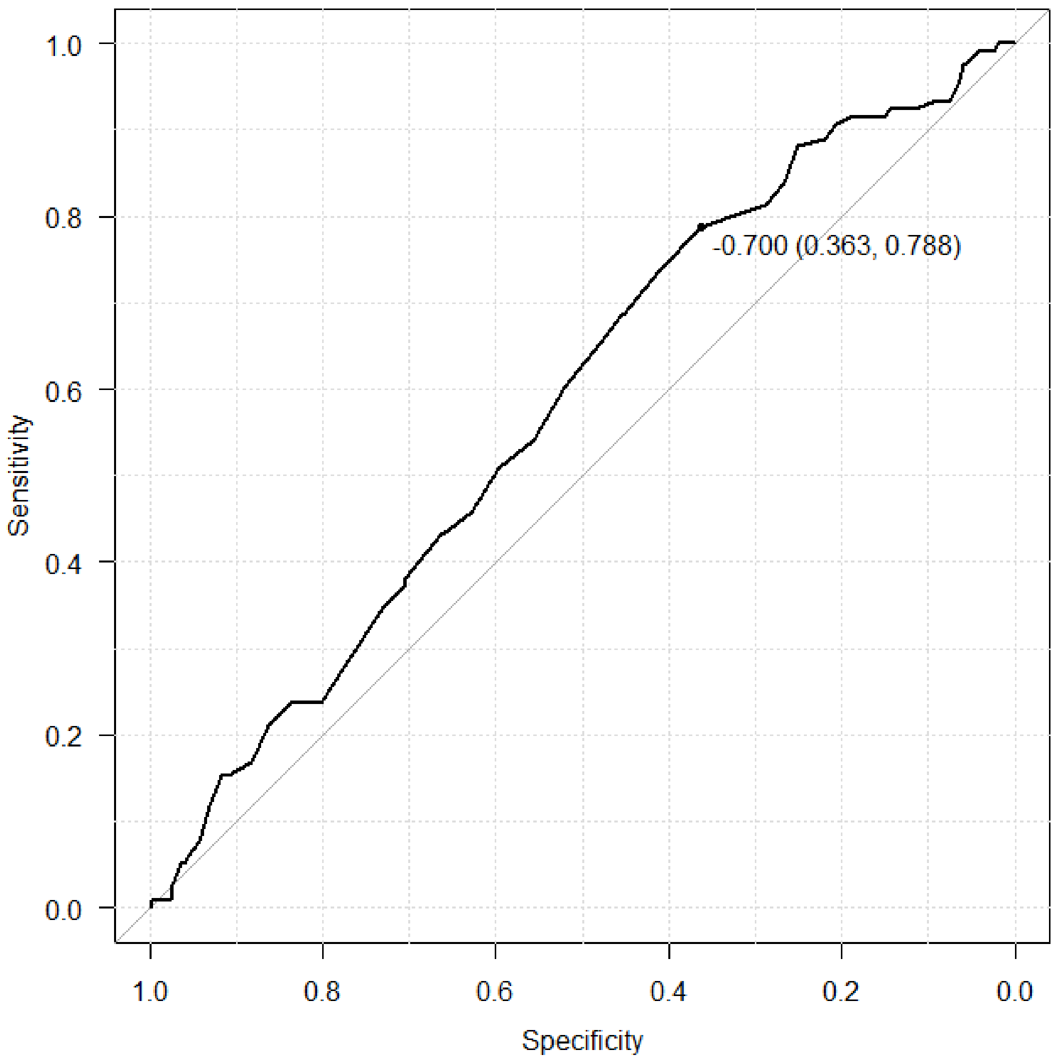

Supplementary Figure 2: ROC curve based on 1st BSEEG score and sepsis.
